# Evaluation of the Antiradical Properties of Phenolic Acids

**DOI:** 10.3390/ijms150916351

**Published:** 2014-09-16

**Authors:** Olga Koroleva, Anna Torkova, Ilya Nikolaev, Ekaterina Khrameeva, Tatyana Fedorova, Mikhail Tsentalovich, Ryszard Amarowicz

**Affiliations:** 1 A. N. Bach Institute of Biochemistry of the Russian Academy of Sciences, Leninsky Prospekt, 33, bld 2, 119071 Moscow, Russia; E-Mails: anna_torkova@mail.ru (A.T.); ilya_mbf@yahoo.com (I.N.); fedorova_tv@mail.ru (T.F.); snowsurfers1@gmail.com (M.T.); 2 Department of Bioengineering and Bioinformatics, Moscow State University, GSP-1, Leninskie Hills, bld 73, 119234 Moscow, Russia; E-Mail: khrameeva@genebee.msu.ru; 3 Institute of Animal Reproduction and Food Research of the Polish Academy of Sciences, Tuwima Street 10, 10-748 Olsztyn, Poland; E-Mail: r.amarowicz@pan.olsztyn.pl

**Keywords:** antioxitant capacity, antioxidant descriptors, quantum-chemical calculations, phenolic acids

## Abstract

Antioxidant capacity (AOC) against peroxyl radical and 2,2'-azino-bis-(3-ethylbenzothiazoline-6-sulphonic acid) diammonium salt (ABTS) radical cation was measured for a series of *p*-hydroxybenzoic (HB) and *p*-hydroxycinnamic (HC) acids at different pH. Quantum-chemical computation was performed using Gaussian 3.0 software package to calculate the geometry and energy parameters of the same compounds. Significant correlations were revealed between AOC and a number of calculated parameters. The most significant AOC descriptors for the studied compounds against peroxyl radical were found to be HOMO energy, rigidity (η) and Mulliken charge on the carbon atom in *m*-position to the phenolic hydroxyl. The most significant descriptor of the antioxidant properties against the ABTS radical cation at pH 7.40 is electron transfer enthalpy from the phenolate ion. The mechanism of AOC realization has been proposed for HB and HC acids against both radicals.

## 1. Introduction

Oxidative stress is one of the general-purpose forms of the body’s response to the exogenic and endogenic factors and plays the significant role in the pathogenesis of inflammatory and dystrophic, neurodegenerative, cardiovascular, oncological diseases and in acceleration of the individual ageing [1,2,3,4,5,6]. Oxidative stress is caused by overproduction of reactive oxygen species (ROS). Exogenic and endogenic antioxidants have a dominant role in the protection of biomolecules from the ROS [7].

A wide range of native and synthetic exogenic antioxidants from food are presently known. Native antioxidants have a number of advantages over synthetic antioxidants, including the lack of side and cumulative effects and lower toxicity.

The carotenoids, thiols, phenolic compounds and peptides are the basic classes of native antioxidants. In spite of the differences in the chemical structures of the antioxidants, the key mechanisms of their interaction with the ROS are hydrogen atom transfer (HAT) or single electron transfer (SET). However, the antioxidant effects of most compounds are realized through a mixed mechanism: either single electron transfer followed by proton transfer (SET–PT), or sequential proton loss and electron transfer (SPLET) [8,9,10,11,12,13,14,15,16,17,18,19,20]. Typically, the resulting intermediates can interact with each other and/or with free radicals in secondary reactions, which contributes to the observed antioxidant effect and causes additional difficulties in analyzing the experimental data.

The quantum-chemical computing methods are an effective tool for examination of the mechanisms of interaction between antioxidants and free radicals and can identify molecular (structural) and electronic descriptors that define the antioxidant properties of different classes of compounds. Recently the use of semiempirical computing methods allowed to reveal the structural features of flavonoids responsible for their antioxidant properties, including the degree of molecule planarity, mutual arrangement of phenolic hydroxyl groups, and the stabilization effects due to the delocalization of the unpaired electron upon the formation of phenoxyl radicals [20,21,22].

When using structural–functional analysis, in parallel with the calculation of the molecular and electronic descriptors the necessary step is to characterize the antioxidant properties of the compounds *in vitro*. At present, over 40 different methods for testing antioxidants *in vitro* are described [23,24,25,26,27,28,29,30,31,32,33,34,35,36,37,38]. Their classification is based on a key mechanism of the interaction of various radicals with tested antioxidants. The widely used methods to characterize natural antioxidants *in vitro* are based on the quenching of the 2,2'-azino-bis-(3-ethylbenzothiazoline-6-sulphonic acid) diammonium salt (ABTS) radical cation (Trolox Equivalent Antioxidant Capacity—TEAC) and peroxyl radical (Oxygen Radical Absorbance Capacity—ORAC). These methods allow for quantitative characterization of the antioxidant properties of compounds with different physicochemical properties and are characterized by high reproducibility and reliability.

The present paper’s general aim is the structural–functional analysis of the phenolic acids in relation to their antioxidant properties, including the investigation of molecular geometry and electronic descriptors for different states of ionization.

Also, the mechanism of antioxidant action against the ABTS radical cation and peroxyl radical has been proposed for phenyl carbonic acids at different pH.

## 2. Results and Discussion

### 2.1. Antiradical Activity of p-Hydroxybenzoic (HB) and p-Hydroxycinnamic (HC)

**Figure 1:**
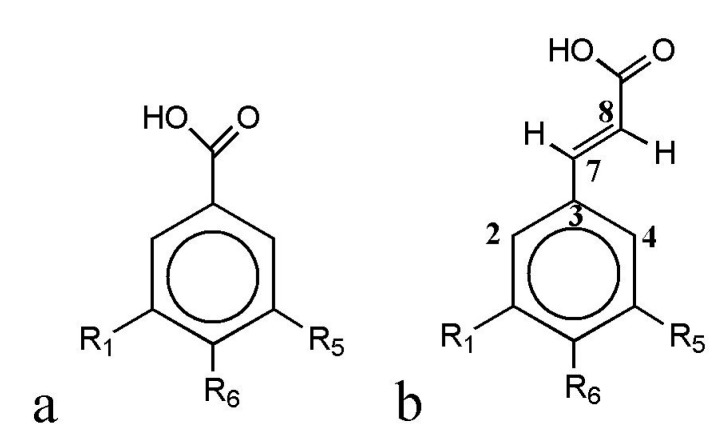
General structures of *p*-hydroxybenzoic (HB, **a**) and *p*-hydroxycinnamic (HC, **b**) acids.

In the present work, *p*-hydroxybenzoic (HB) and *p*-hydroxycinnamic (HC) acids with various substituents (Figure 1, Table 1) were chosen as the objects of study. *p*-Hydroxybenzoic (*p*-HBA), vanillic (VA) and syringic acid (SyrA) are structural analogs of HC acids: *p*-coumaric (CA), ferulic (FA) and sinapic (SinA) ones.

Antioxidant capacity (AOC) of HB and HC acids was determined against the ABTS radical cation (TEAC assay) and peroxyl radical (ORAC assay) at pH 7.4 (Table 2).

The AOC of the studied HB and HC acids is, on the average, three times higher than that of d,l-α-tocopherol, and 3.0 and 5.7 times higher than AOC of l-ascorbic acid against the ABTS radical cation and peroxyl radical, respectively (Table 2).

**Table 1:** Substituents in the HB and HC acid structures.

Phenolic Acids	R_1_	R_5_	R_6_
*p*-Hydroxybenzoic (HB) acids:			
*p*-Hydroxybenzoic (HBA)	H	H	OH
Vanillic (VA)	H	OCH_3_	OH
Syringic (SyrA)	OCH_3_	OCH_3_	OH
Gallic acid (GalA)	OH	OH	OH
*p*-Hydroxycinnamic (HC) acids:			
*p*-Coumaric (CA)	H	H	OH
Ferulic (FA)	H	OCH_3_	OH
Sinapic (SinA)	OCH_3_	OCH_3_	OH

The positions of the substituents in the benzene ring are numbered according to Figure 1.

HC acids are characterized by 1.2–2.5 times higher AOC values against both types of radicals compared to the corresponding HB acids (Table 2). Such high values are explained by the stabilization of phenoxyl radicals of HC acids due to the delocalization of the unpaired electron in case of benzene ring conjugation with C3 chain, and less pronounced influence of the carboxyl group, notable for its negative inductive and mesomeric effects, on the distribution of electron density in the benzene ring due to the presence of ethenyl bridge.

**Table 2:** Antioxidant capacity of HB and HC acids against the 2,2'-azino-bis-(3-ethylbenzothiazoline-6-sulphonic acid) diammonium salt (ABTS) radical cation (Trolox Equivalent Antioxidant Capacity—TEAC, 50 µM PBS, pH 7.4) and peroxyl radical (Oxygen Radical Absorbance Capacity—ORAC, 75 µM Na-phosphate buffer, pH 7.4).

Phenolic Acids	TEAC (μmol TE/μmol)	ORAC (μmol TE/μmol)
*p*-Hydroxybenzoic (HBA)	2.64 ± 0.07	2.17 ± 0.18
Vanillic (VA)	2.63 ± 0.08	3.44 ± 0.19
Syringic (SyrA)	1.46 ± 0.09	1.52 ± 0.11
Gallic acid (GalA)	5.76 ± 0.21	1.08 ± 0.05
*p*-Coumaric (CA)	3.04 ± 0.18	5.02 ± 0.26
Ferulic (FA)	3.90 ± 0.21	4.59 ± 0.21
Sinapic (SinA)	3.66 ± 0.15	2.94 ± 0.19
l-Ascorbic acid	1.01 ± 0.02	0.52 ± 0.04
d,l-α-Tocoferol ^a^	1.00 ± 0.02	1.01 ± 0.06

^a^ To increase the d,l-α-tocoferol solubility in aqueous medium, it was dissolved in an acetone–water (1:1, *v*/*v*) mixture containing 7% of methyl-β-cyclodextrine with methylation degree of 10%–12%.

Experimentally determined the AOC values of HB and HC acids against peroxyl radical are in a good agreement with literature data [39,40]. Specifically, experimental and earlier published values of AOC against peroxyl radical for VA were 3.44 ± 0.19 and 3.21 ± 0.14 µmol TE/µmol [41]; for CA—5.02 ± 0.26 and 4.47 ± 0.21 µmol TE/µmol [42]; for GA—1.08 ± 0.05 and 1.05–1.64 µmol TE/µmol [43,44]; for FA—4.59 ± 0.21 and 3.88–4.47 µmol TE/µmol [41,42], respectively. In contrast to the above phenolic acids, the obtained AOC values for SinA are 1.8 times higher than those described in the work of Nenadis *et al*. [43]. For *p*-HBA and SyrA the AOC values against peroxyl radical determined by ORAC method with use of fluorescein as an oxidation substrate were originally defined in the present work. It should be noted that the *in vitro* antiradical behavior of the studied compounds can be different from that explicated *in vivo*. Either synergic or infra-additive effects can occur when the antioxidant compounds are studied in living systems. The adding of propolis, for instance, substantially alters the antiradical properties of essential oil, although propolis itself exhibits almost zero antioxidant activity in FRAP (Ferric Reducing Antioxidant Power) and TEAC assays [45].

The AOC values of HC acids against peroxyl radical (Table 2) decreases in the row CA > FA > SinA, which is consistent with the AOC analysis for HB and HC acids by ORAC method with use of β-phycoerythrin protein as a fluorescent oxidation substrate [46]. Thus, the introduction of a methoxy substituent in the *O*-position to the 4-hydroxy group leads to a decrease in AOC of HC acids against peroxyl radical, which can be explained by a decrease of electron density in the benzene ring and a reduced interaction efficiency of HC acids with electrophilic particles.

In contrast to HC acids, the introduction of one methoxy group in the *O*-position to the *p*-hydroxy group increases the AOC of HB acids, but the introduction of the second methoxy substituent reduces the AOC. The increase of AOC of HB acids having a methoxy group in *O*-position to the phenolic hydroxyl is caused by a more effective stabilization of the formed phenoxyl radical.

The AOC values of HB acids against peroxyl radical decrease in a row VA > *p*-HBA > SyrA > GalA (Table 2), which conflicts with the data of Yen *et al*. (GalA > *p*-HBA > SyrA > VA) [46]. It may be caused by using different fluorescent markers in ORAC analysis. This dissimilarity confirms that the structure-functional assay for HB and HC acids should only be carried out in an invariable experimental model. This can be illustrated by literature data on AOC of phenolic acids against the ABTS radical cation. The AOC values of phenolic acids vary considerably depending on the ABTS radical cation generation system (enzymatic/non-enzymatic), time of analysis and solvent system. The overall trend is an increase in AOC of phenolic acids upon incubation time prolongation and increase of the polarity and pH of reaction medium [47,48].

**Figure 2:**
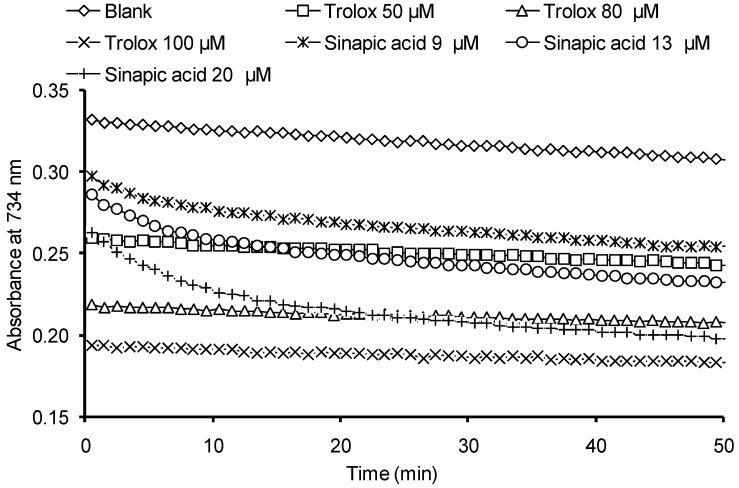
Kinetic curves describing the optical density decrease at interaction of the ABTS radical cation solution with various antioxidants.

One of the limitations of the TEAC method is that AOC is determined at a fixed duration of reaction of antioxidants with the ABTS radical cation (usually 1–10 min). As a result, the stationary phase in kinetics of interaction of phenolic antioxidants with the ABTS radical cation is often not achieved, which naturally results in underestimated AOC values. Today, two stages are differentiated in the kinetics of interaction of the ABTS cation–radical with phenolic antioxidants: the fast stage and the slow one [49,50]. The duration of the fast stage is 0.1 s, while the duration of the slow one is determined by the structure of an antioxidant and can be up to several hours. Thus, to properly quantify the AOC of HB and HC acids against the ABTS radical cation it appears necessary to determine the optimal reaction monitoring time range, in which the kinetics of the reaction for all the compounds involved would achieve the stationary phase. Examples of kinetic curves are shown in Figure 2 describing the optical density decrease at interaction of Trolox and SinA with the ABTS cation–radical. For the rest of the investigated compounds the dependences obtained were similar to the SinA.

Trolox is characterized by mono-phase kinetics of interaction with the ABTS radical cation, whereas for the studied HB and HC acids the two-phase kinetics is typical, the stationary phase being achieved after 30–40 min of the reaction (Figure 2). Thus, the AOC of phenolic acids against the ABTS radical cation is determined at 40th min of reaction.

The studied hydroxybenzoic and hydroxycinnamic acids have been previously found in saffron [51], coffee [41], olive oil [52] and other antioxidant natural sources. The obtained AOC values of HB and HC acids against the ABTS radical action were compared to the earlier published data (Table 3). For all investigated compounds with the exception of SirA, the AOC values defined in this paper were 1.2–1.6 times higher than those in literature, with the largest differences observed for sinapic acid due to the increase in reaction time.

**Table 3:** Comparison of the experimental AOC of phenyl carbonic acids against the ABTS radical cation with literature data.

Phenolic Acid	TEAC (μmol TE/μmol)				
*p*-HBA	2.64 ± 0.07	2.64 ± 0.07	–	–	–
VA	2.63 ± 0.08	1.8	–	1.52 ± 0.01	1.42 ± 0.3
SyrA	1.46 ± 0.09	1.6	–	–	1.40 ± 0.03
GalA	5.76 ± 0.21	4.3	–	–	2.15 ± 0.01
CA	3.04 ± 0.18	2.3	2.39 ± 0.09	–	2.27 ± 0.01
FA	3.90 ± 0.21	3.2	1.97 ± 0.02	2.32 ± 0.09	1.87 ± 0.06
SinA	3.66 ± 0.15	2.2	2.09 ± 0.11	–	–
Conditions of analysis	PBS, pH 7.40, 40th min	PBS, pH 7.40, 1st min	PBS, pH 7.40, 6th min	PBS, pH 7.40, 10th min	ethanol, 1st min
Data from	Experiment	[46]	[53]	[41]	[54]

### 2.2. Influence of pH on Antioxidant Capacity (AOC)

It is known that the antioxidant action efficiency of HB and HC acids depends on the ionization degree of carboxyl group and phenolic hydroxyl. The ionization degree affects the distribution of electronic density in aromatic system. In particular, the increase of AOC against the ABTS radical cation–radical with increasing pH has been shown for a number of flavonoids and fluorinated *p*-hydroxybenzoic acids [22,47].

**Figure 3:**
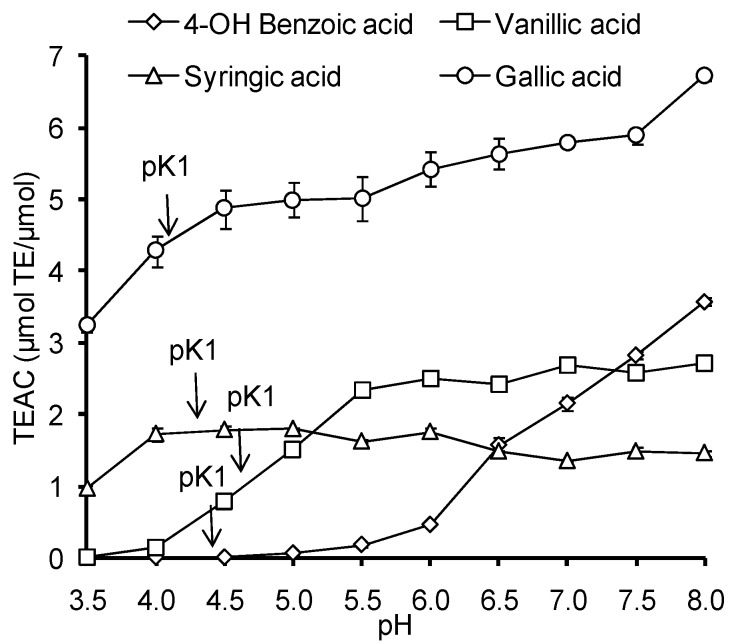
pH-Dependence of AOC against the ABTS radical cation for HB and HC acids.

The influence of pH in the range of 3.5–8.0 on the AOC against the ABTS radical cation was studied for HB and HC acids (Figure 3). In this pH range the ionization of carboxyl groups of HB and HC acids takes place, (pKa 4.10–4.58), and the ionization of phenolic hydroxyl begins in the case of GalA and SinA (p*K*_phenol_ 8.90–8.94).

The AOC values of standard antioxidant—Trolox—in the investigated range of pH did not change despite the ionization of carboxyl group in a chromane ring (p*K* = 3.89), due to the spatial remoteness of carboxyl and phenolic groups, lack of conjugation between benzene and chromane rings and high p*K* value of trolox phenolic hydroxyl (p*K* = 11.80) [55].

Unlike Trolox, in all studied HB and HC acids the values of AOC increased with increasing pH, however, the dynamics and the intensity of that growth were individual for each compound. It was observed that *p*-HBA and VA do not react with the ABTS radical cation at low pH, and exhibit the most pronounced growth of the AOC values with increasing pH (Figure 3). For most acids (with the exception of *p*-HBA), a pronounced growth of AOC value was observed in the range of carboxyl group ionization (4.0–5.5). It should be noted that the features of pH dependence of gallic acid AOC against the ABTS radical cation are in good agreement with the data of Lemanska *et al* [22] for pH range of 5.5 to 8.0.

**Figure 4:**
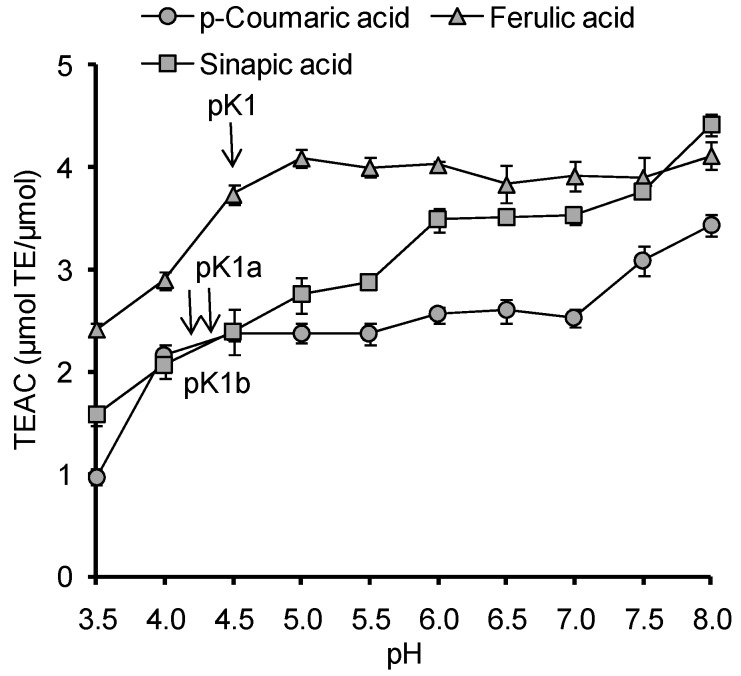
pH-Dependence of AOC against the ABTS radical cation for HC acids (pK1a is sinapic acid related, pK1b is p-coumaric acid related).

The analysis of pH-dependencies of AOC for all investigated HB and HC acids, with the exception of *p*-HBA, indicates that the carboxyl group deprotonation results in 1.7–2.4 times increase of their AOC against the ABTS radical cation (Figure 3 and Figure 4). At pH > 7.5 the deprotonation of phenolic hydroxyl begins, accompanied by a change of the antioxidant mechanism of phenolic acids the corresponding phenolate ions provide direct single electron transfer to the ABTS radical cation, which leads to an increase of AOC at pH 8.0 for GalA (p*K*_phenol_ = 8.90), sinapic acid (p*K*_phenol_ = 8.94), CA (p*K*_phenol_ = 9.21) and, to a lesser extent, for FA (p*K*_phenol_ = 9.21). For SirA and VA the p*K* values representing the phenolic hydroxyl dissociation shifted to a more alkaline region (9.49 and 9.39 respectively), so the increase of AOC at pH 8.0 was not observed. Thus, the dissociation processes of ionogenic groups in HB and HC acids have a significant impact on their antioxidant properties. That is why for the further research of molecular and electronic descriptors, as well as for discrimination of antioxidant mechanisms of HB and HC acids, the quantum-chemical calculations were carried out for different states of ionization.

### 2.3. Computation of Molecular and Electronic Descriptors for Antioxidant Properties of HB and HC Acids

Earlier studies [8,9,10,11,12,13,14,15,16,17,18] indicate that free radicals can be deactivated by phenolic antioxidants according to three possible mechanisms:

- Hydrogen atom transfer (HAT):
ArOH + X· → ArO· + XH
(1)
- Sequential proton loss and electron transfer (SPLET):
ArOH → ArO^−^ + H^+^ (2)
ArO^−^ + X· + H^+^ → ArO· + XH (3)
- Single electron transfer followed by proton transfer (SET–PT):
ArOH + X· → ArOH^+^ + X^−^ (4)
ArOH + X^−^ → ArOH· + XH (5)

To discriminate the mechanism of radical deactivation by phenyl carbonic acids, the influence of various molecular and electronic descriptors on their antioxidant properties was studied.

### 2.4. Spin Density and Electrophilic Attack of Radicals

The calculated Mulliken charges on the carbon atoms in the benzene ring, as well as oxygen and hydrogen atoms in phenolic hydroxyl and metoxy groups are presented in Table 4. The atoms with the highest electronic density values are known to be sensitive to free-radical attacks [8]. According to the Table 4a,c, in the studied compounds the carbon atoms most preferable for electrophilic agent attacks are those located in *m*-positions (C_2_, C_4_) to the phenolic hydroxyl. For VA, SyrA, *cis*-CA, *trans*-CA, *trans*-FA or the corresponding hydroxybenzoates/hydroxycinnamates such preferred position is C_2_.

**Table 4:** (**a**) Mulliken charges at carbon atoms of aromatic systems of HB acids, their radicals and cation–radicals in various states of ionization; (**b**) Mulliken charges at oxygen and some hydrogen atoms of HB acids, their radicals and cation–radicals in various states of ionization; (**c**) Mulliken charges at carbon atoms of aromatic systems of HC acids, their radicals and cation–radicals in various states of ioni*z*ation; (**d**) Mulliken charges at oxygen and some hydrogen atoms of HB acids, their radicals and cation–radicals in various states of ionization (numbering of atoms according to Figure 1).

**(a)**							
**Phenolic Acid**	**State of Ionization**	**C_3_**	**C_4_**	**C_5_**	**C_6_**	**C_1_**	**C_2_**
*p-*OH Benzoic	*z* = 0	0.489	−0.133	−0.440	−0.119	−0.003	−0.434
	*z* = −1	1.099	−0.838	0.393	−.0.890	0.468	−0.885
	*z* = −2	0.932	−0.619	0.314	−1.108	0.314	−0.619
	radical, *z* = 0	1.037	−0.832	0.455	−0.797	0.515	−0.915
	radical, *z* = −1	1.151	−0.938	0.535	−0.838	0.535	−0.938
	radical cation, *z* = −1	1.091	−0.827	0.535	−0.774	0.610	−1.118
	radical cation, *z* = 0	1.301	−0.909	0.520	−0.809	0.446	−0.190
Vanillic	*z* = 0	0.569	0.518	−0.455	−0.633	0.194	−0.775
	*z* = −1	0.590	0.379	−0.585	−0.410	−0.104	−0.371
	*z* = −2	0.763	−0.176	−0.375	−0.513	0.164	−0.597
	radical, *z* = 0	0.853	0.155	−0.325	−0.664	0.409	−0.872
	radical, *z* = −1	0.931	−0.078	−0.287	−0.622	0.501	−0.858
	radical cation, *z* = −1	0.704	−0.302	−0.398	−0.301	−0.112	−0.268
	radical cation, *z* = 0	0.782	0.335	−0.497	−0.324	−0.062	−0.446
Syringic	*z* = 0	1.481	−0.149	−0.456	−0.504	0.0552	−0.328
	*z* = −1	−0.240	0.278	0.120	−0.038	0.142	−0.278
	*z* = −2	0.547	−0.040	−0.794	0.516	−.0793	−0.042
	radical, *z* = 0	1.243	−0.201	−0.478	−0.386	−0.380	−0.303
	radical, *z* = −1	1.249	−0.071	−0.459	−0.498	−0.459	−0.071
	radical cation, *z* = −1	1.513	−0.099	−0.421	−0.475	−0.534	−0.286
	radical cation *z* = 0	0.412	0.045	0.050	0.001	0.252	−0.035
Gallic	*z* = 0	0.859	−0.376	−0.371	−0.166	−0.219	−0.152
	*z* = −1	0.910	−0.262	−0.425	−0.127	−0.225	−0.237
	radical cation, *z* = −1	0.867	−0.034	0.204	−0.107	−0.309	−0.426
	radical cation, *z* = 0	1.113	−0.256	−0.168	−0.138	−0.391	−0.234
	radical C_1_, *z* = 0	0.843	−0.113	−0.446	0.233	0.556	0.154
	radical C_6_, *z* = 0	0.788	−0.147	−0.354	−0.079	−0.350	−0.274
	radical C_5_, *z* = 0	0.900	−0.185	−0.450	0.250	−0.619	−0.182
	radical C_1_, *z* = 0	0.796	−0.111	−0.502	0.284	−0.637	−0.069
	radical C_6_, *z* = −1	0.863	−0.184	−0.334	−0.139	−0.334	−0.184
	radical C_5_, *z* = −1	0.900	−0.114	−0.506	0.301	−0.696	−0.108
	*z* = −2, C_1_	0.758	−0.192	−0.642	0.343	−0.735	−0.090
	*z* = −2, C_6_	0.695	0.040	−0.486	−0.048	−0.655	−0.145
	*z* = −2, C_5_	0.758	−0.09	−0.735	0.343	−0.641	−0.192
	*z =* −3, C_1_, C_5_	0.549	0.006	−1.006	0.487	−0.863	0.072
	*z =* −3, C_1_, C_6_	1.599	−3.920	2.661	1.152	−0.608	−0.329
	*z =* −3, C_6_, C_5_	1.598	−0.329	−0.609	1.150	3.664	−3.924
**(b)**							
**Phenolic Acid**	**State of Ionization**	**O(C_6_)**	**O(C_5_)**	**O(C_1_)**	**H [O(C_6_)]**	**H [O(C_5_)]**	**H [O(C_1_)]**
*p-*OH Benzoic	*z* = 0	−0.223	–	–	0.273	–	–
	*z* = −1	−0.269	–	–	0.242	–	–
	*z* = −2	−0.559	–	–	–	–	–
	radical, *z* = 0	−0.233	–	–	–	–	–
	radical, *z* = −1	−0.350	–	–	–	–	–
	radical cation, *z* = −1	−0.046	–	–	0.321	–	–
	radical cation, *z* = 0	−0.209	–	–	0.268	–	–
Vanillic	*z* = 0	−0.239	−0.262	–	0.295	–	–
	*z* = −1	−0.296	−0.281	–	0.276	–	–
	*z* = −2	−0.555	−0.194	–	–	–	–
	radical, *z* = 0	−0.250	−0.106	–	–	–	–
	radical, *z* = −1	−0.341	−0.134	–	–	–	–
	radical cation, *z* = −1	−0.094	−0.180	–	0.332	–	–
	radical cation, *z* = 0	−0.233	−0.258	–	0.298	–	–
Syringic	*z* = 0	−0.253	−0.267	−0.172	0.304	–	–
	*z* = −1	−0.568	−0.461	−0.375	0.404	–	–
	*z* = −2	−0.579	−0.185	−0.185	–	–	–
	radical, *z* = 0	−0.253	−0.122	−0.121	–	–	–
	radical, *z* = −1	−0.329	−0.149	−0.149	–	–	–
	radical cation, *z* = −1	−0.099	−0.242	−0.096	0.347	–	–
	radical cation *z* = 0	−0.500	−0.447	−0.353	0.424	–	–
Gallic	*z* = 0	−0.240	−0.232	−0.306	0.290	0.270	0.288
	*z* = −1	−0.297	−0.273	−0.337	0.270	0.259	0.274
	radical cation, *z* = −1	−0.078	−0.258	−0.125	0.341	0.329	0.318
	radical cation, *z* = 0	−0.232	−0.301	−0.227	0.293	0.289	0.274
	radical C_1_, *z* = 0	−0.181	−0.300	−0.239	0.294	0.286	–
	radical C_6_, *z* = 0	−0.247	−0.176	−0.175	–	0.271	0.268
	radical C_5_, *z* = 0	−0.177	−0.306	−0.230	0.295	–	0.273
	radical C_1_, *z* = −1	−0.237	−0.330	−0.306	0.273	0.271	–
	radical C_6_, *z* = −1	−0.332	−0.220	−0.220	–	0.254	0.254
	radical C_5_, *z* = −1	−0.235	−0.374	−0.271	0.274	–	0.257
	*z* = −2, C_1_	−0.352	−0.311	−0.592	0.242	0.222	–
	*z* = −2, C_6_	−0.613	−0.320	−0.295	–	0.235	0.211
	*z* = −2, C_5_	−0.352	−0.592	−0.311	0.242	–	0.222
	*z* = −3, C_1_, C_5_	−0.382	−0.660	−0.613	0.191	–	–
	*z* = −3, C_1_, C_6_	−0.515	−0.486	−0.515	–	−1.179	–
	*z* = −3, C_6_, C_5_	−0.515	−0.515	−0.486	–	–	−1.179
**(c)**							
**Phenolic Acid**	**State of Ionization**	**C_3_**	**C_4_**	**C_5_**	**C_6_**	**C_1_**	**C_2_**
*cis*-Caffeic	*z* = 0	0.809	0.029	−0.410	−0.296	0.027	−1.045
	*z* = −1	1.331	0.245	−0.386	−0.328	−0.009	−1.293
	*z* = −2	1.767	0.231	−0.163	−0.860	−0.008	−1.315
	radical, *z* = 0	1.352	0.164	−0.140	−0.400	0.000	−1.195
	radical, *z* = −1	1.706	−0.885	0.312	−0.729	0.308	−0.884
	radical cation, *z* = −1	1.545	−1.342	0.081	−0.303	−0.385	0.277
	radical cation, *z* = 0	1.537	−1.409	0.093	−0.422	0.379	0.230
*trans*-Caffeic	*z* = 0	1.316	0.342	−0.319	−0.481	−0.099	−1.262
	*z* = −1	1.193	0.320	−0.327	−0.497	−0.071	−1.258
	*z* = −2	1.549	−0.520	0.393	−1.076	0.247	−1.356
	radical, *z* = 0	1.272	0.230	−0.141	−0.487	−0.257	−1.042
	radical, *z* = −1	1.314	0.012	0.047	−0.648	−0.070	−1.297
	radical cation, *z* = −1	1.420	0.260	−0.101	−0.382	−0.410	−1.053
	radical cation, *z* = 0	1.272	−1.287	−0.102	−0.484	−0.320	−0.355
*cis*-Ferulic	*z* = 0	1.352	−1.094	0.018	0.088	−0.517	0.061
	*z* = −1	1.626	−1.456	−0.041	0.123	−0.644	0.138
	*z* = −2	1.625	−1.188	−0.182	−0.258	−0.326	−0.012
	radical, *z* = 0	1.481	0.054	−0.329	−0.006	0.152	−1.425
	radical, *z* = −1	1.231	−1.162	−0.238	0.264	−0.083	−0.093
	radical cation, *z* = −1	1.589	−1.724	0.350	−0.431	−0.668	0.570
	radical cation *z* = 0	1.896	−1.503	0.002	0.159	−0.599	−0.015
*trans*-Ferulic	*z* = 0	1.710	0.107	−0.285	−0.453	−0.401	−0.948
	*z* = −1	1.488	0.173	−0.706	−0.046	0.018	−1.381
	*z* = −2	1.424	−0.170	−0.125	−0.298	−0.200	−0.116
	radical, *z* = 0	1.136	−0.652	−0.640	−0.396	0.109	−0.181
	radical, *z* = −1	1.542	0.042	−0.−23	−0.297	−0.169	−1.250
	radical cation, *z* = +1	1.377	−0.639	−0.519	−0.551	−0.014	−0.107
	radical cation *z* = 0	1.683	0.083	−0.254	−0.439	−0.426	−0.930
*cis*-Sinapic	*z* = 0	1.564	−0.739	−0.477	−0.093	−0.368	0.042
	*z* = −1	2.014	−0.856	−0.739	0.027	−0.226	−0.337
	*z* = −2	1.411	−1.014	−0.219	0.011	−0.289	−0.044
	radical, *z* = 0	1.454	−0.262	−0.592	−0.353	0.152	−0.334
	radical, *z* = −1	1.740	−0.754	−0.883	−0.112	0.162	−0.146
	radical cation, *z* = −1	1.475	−0.495	−0.094	−0.707	−0.185	−0.083
	radical cation, *z* = 0	1.662	0.164	−0.490	−0.63	−0.537	−0.630
*trans*-Sinapic	*z* = 0	1.384	−0.670	−0.384	−0.636	−0.352	0.230
	*z* = −1	1.339	−0.809	−0.407	−0.645	−0.375	0.229
	*z* = −2	1.295	−0.230	−0.038	0.391	−0.483	−1.216
	radical, *z* = 0	1.648	−0.180	−0.357	−0.373	−0.564	−0.742
	radical, *z* = −1	1.543	−0.218	0.277	−0.212	−0.715	−0.898
	radical cation, *z* = −1	1.761	−0.092	−0.139	−0.666	−0.843	−0.425
	radical cation, *z* = 0	1.368	−0.579	−0.407	−0.619	−0.375	−0.253
**(d)**							
**Phenolic Acid**	**State of Ionization**	**O(C_6_)**	**O(C_5_)**	**O(C_1_)**	**H [O(C_6_)]**	**H [O(C_5_)]**	**H [O(C_1_)]**
*cis*-Caffeic	*z* = 0		–	–	0.268	–	–
	*z* = −1		–	–	0.253	–	–
	*z* = −2		–	–	–	–	–
	radical, *z* = 0		–	–	–	–	–
	radical, *z* = −1		–	–	–	–	–
	radical cation, *z* = −1		–	–	0.315	–	–
	radical cation, *z* = 0		–	–	0.271	–	–
*trans*-Caffeic	*z* = 0		–	–	0.271	–	–
	*z* = −1		–	–	0.254	–	–
	*z* = −2		–	–	–	–	–
	radical, *z* = 0		–	–	–	–	–
	radical, *z* = −1		–	–	–	–	–
	radical cation, *z* = −1		–	–	0.310	–	–
	radical cation, *z* = 0		–	–	0.273	–	–
*cis*-Ferulic	*z* = 0		–	−0.162	0.276	–	–
	*z* = −1		–	−0.168	0.262	–	–
	*z* = −2		–	−0.200	–	–	–
	radical, *z* = 0		–	−0.175	–	–	–
	radical, *z* = −1		–	−0.367	–	–	–
	radical cation, *z* = −1		−1.724	−0.086	0.315	–	–
	radical cation *z* = 0		−1.503	0.154	0.280	–	–
*trans*-Ferulic	*z* = 0		0.107	–	0.296	–	–
	*z* = −1		0.173	–	0.263	–	–
	*z* = −2		−0.170	–	–	–	–
	radical,*z* = 0		−0.652	–	–	–	–
	radical, *z* = −1		0.042	–	–	–	–
	radical cation, *z* = −1		−0.639	–	0.335	–	–
	radical cation *z* = 0		0.083	–	0.298	–	–
*cis*-Sinapic	*z* = 0		−0.739	−0.163	0.308	–	–
	*z* = −1		−0.856	−0.170	0.296	–	–
	*z* = −2		−1.014	−0.195	–	–	–
	radical, *z* = 0		−0.262	−0.199	–	–	–
	radical, *z* = −1		−0.754	−0.180	–	–	–
	radical cation, *z* = −1		−0.495	−0.123	0.342	–	–
	radical cation, *z* = 0		0.164	−0.152	0.311	–	–
*trans*-Sinapic	*z* = 0		−0.670	−0.174	0.305	–	–
	*z* = −1		−0.809	−0.187	0.291	–	–
	*z* = −2		−0.230	−0.177	–	–	–
	radical, *z* = 0		−0.180	−0.270	–	–	–
	radical, *z* = −1		−0.218	−0.145	–	–	–
	radical cation, *z* = −1		−0.092	−0.124	0.344	–	–
	radical cation, *z* = 0		−0.579	−0.171	0.308	–	–

For GalA, *cis*-SinA, and *trans*-SinA in both states of ionization the preferred position for electrophilic attack is C_4_. For GalA and *p*-HBA with ionized carboxyl groups electrophilic attacks could equally well be aimed at both positions C_2_ and C_4_. In the case of *cis*- and *trans*-isomers of HC acids and *p*-HBA the deprotonation of carboxyl group leads to 1.1–1.4 times increase of the electron density on the carbon atom preferred for electrophilic attacks (Table 4a,c). On the contrary to the HC acids, for GalA, SyrA and VA the deprotonation of carboxyl group was noted to reduce the spin density at C_4_ (GalA) and C_2_ (SyrA, VA) positions.

**Figure 5:**
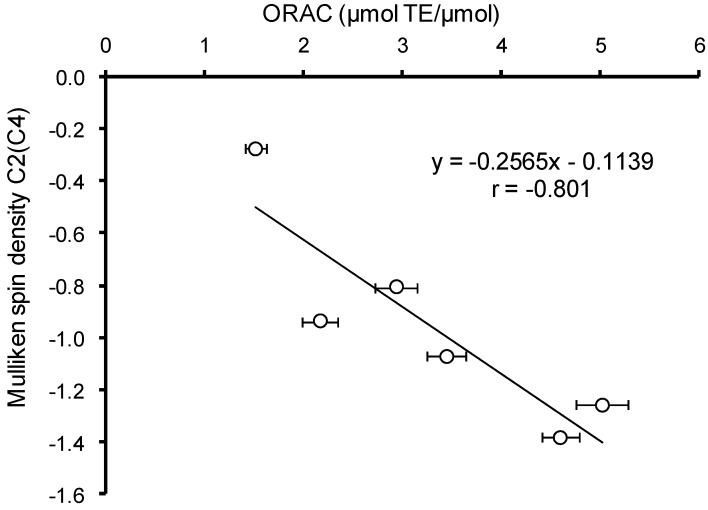
Correlation analysis between AOC of hydroxyaromatic acids against peroxyl radical and Mulliken charge at carbon atom preferred for electrophilic agent attacks.

The increase of the electron density was expected to contribute to the efficiency of the primary interaction of hydroxyaromatic acids with free radicals and other electrophilic particles. To confirm this assumption was performed the correlation analysis between Mulliken charge values on the carbon atom preferable for electrophilic compound attacks and AOC values of HB and HC acids against the ABTS radical cation and peroxyl radical. In the case of ABTS no significant correlation was revealed for various states of ionization, due to the uncompetitive nature of this analysis method and the existence of a large number of secondary reactions, contributing to the AOC value. In the case of peroxyl radical, the experiments were carried out at pH 7.40, thus, the correlation analysis was carried out only for monoanion forms of HB and HC acids. A significant (*p* < 0.05) inverse correlation (*r* = −0.801) between the Mulliken charge and AOC against peroxyl radical was observed (Figure 5). The exception of VA from the analysis arrays increases the value of the Spearman correlation coefficient to 0.925 without change of correlation significance.

HB acids are characterized by higher values of Mulliken charge on the carbon atom in *meta*-position to the phenolic hydroxyl compared to their structural analogues among HC acids, which correlates with the previously identified differences in the values of their AOC against peroxyl radical (Table 2).

In addition to the changes in the electron density at C_2_/C_4_ position in the benzene ring, the carboxyl groups, deprotonation results in reduction of O–H bond polarity and facilitates the hydrogen atom transfer. Thus, the sum of Mulliken charges for oxygen and hydrogen atoms of phenolic hydroxyl in non-ionized form of HB and HC acids varies within 0.050–0.056 and 0.032–0.056, respectively (Table 4b,d). For the relevant benzoates and cinnamates the sum of Mulliken charges for oxygen and hydrogen atoms of phenolic hydroxyl reduced to (−0.164)–(−0.020) and (−0.024)–(−0.001).

**Figure 6:**
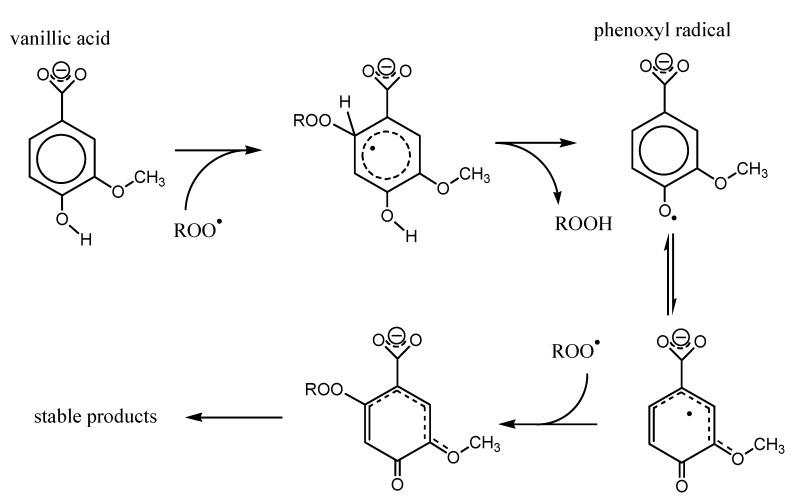
Mechanism of peroxyl radical interaction with *p*-HB and *p*-HC acids (Vanillic (VA) as an example).

Based on the data obtained, the scheme was suggested that describes the mechanism of interaction of *p*-HB and *p*-HC acids (VA as an example) with peroxyl radical (Figure 6). The primary attack by the peroxyl radical aims the aromatic system at a carbon atom with the largest electronic density (lowest Mulliken charge), which is C_2_ in the case of VA, with further hydrogen atom abstraction and formation of hydroperoxide ROOH and phenoxyl radical. Further the delocalization of the unpaired electron leads to the formation of the most thermodynamically stable form of phenoxyl radical. In case of vanillic acid, maximum electron density is localized on C_2_ carbon atom, where the secondary peroxyl radical attack aims, followed by further chemical transformations yielding the stable products.

As shown in Figure 6, the presence of the substituents in *ortho*-positions to the phenolic hydroxyl can stabilize the resulting phenoxyl radicals, reducing the probability of recombination and increasing the efficiency of secondary reactions with peroxyl radical. This correlates with the increase of the AOC of phenolic antioxidants.

As follows from the data in Table 4a–d, the stabilization effect was ascertained for radicals and radical cations of VA, SyrA, *cis*-/*trans*-FA and *cis*-/*trans*-SinA with non-ionised carboxyl group. However, the introduction of substituents with negative inductive effect in the benzene ring creates a steric barrier for interaction with bulk radicals, and also variants the electron density in the benzene ring.

Thus, AOC of HB and HC acids depends on the nature and positions of substituents, namely:

(1) The influence of substituents on Mulliken charge on carbon atoms in C_2_/C_4_ positions.
(2) The stabilization effects due to delocalization of the unpaired electron in phenoxyl radicals.
(3) The steric effects of the substituents.

A following trend is observed when comparing the *trans*-CA and *trans*-SinA: the introduction of two metoxy groups leads to a decrease of the electron density in C_4_ position and creates steric barriers, which in combination results in 1.7 times lower *trans*-SinA AOC against peroxyl radical compared to *trans*-CA. Introduction of 1 metoxy group in the benzene ring (*trans*-FA) leads only to a minor (1.1 times) reduction of AOC against peroxyl radical, correlating with an insignificant increase of the spin density at C_2_ carbon atom of the benzene ring.

### 2.5. Structural Descriptors and Stabilizzation of the Phenoxyl Radical

The stabilization mechanism in HC acids can involve, besides metoxy groups, the C_3_ chain, its conjugation degree with the benzene ring being dependent on their relative positions. Such stabilization effects are considered to cause higher values of AOC in HC acids compared to their structural analogues among HB acids [54], which is confirmed by results of the quantum-chemical calculations (Table 2). It was observed that the side chain participates in delocalization of unpaired electrons in *cis*-isomers of FA and SinA during the formation of a radical with a deprotonated carboxyl group. The side chain of *trans*-isomers of HC acids participate in delocalization of unpaired electrons in case of radicals and radical cations corresponding to the uncharged acid form, and also a radical of monoanion form.

The maximum degree of conjugation between the benzene ring and the side chain in HC acids will be achieved when the carbon atoms in the benzene ring and the double bond in C_3_ chain share one plane. To estimate the possibility of π–π conjugation of the carbon atoms in the benzene ring with C3 side chain for different states of ionization of *cis*- and *trans*-isomers of HC acids, the values of dihedral angles between 4, 3, 7, 8 and 2, 3, 7, 8 carbon atoms were calculated (Table 5). The analysis of the data in Table 5 shows that the delocalization of the unpaired electron in the phenoxyl radicals of HC acids is possible when the angle between the planes of the benzene ring and C3 side chain is no more than ±10°. Thus, such delocalization is impossible in cation–radicals of *cis*-isomers of HC acids and uncharged radicals of HC acids. At the same time, in the case of *trans*-isomers there is a possibility of electron delocalization in phenoxyl radicals and radical cations in all states of ionization of HC acids, which increases their stability. Apparently, it is the greater stability of HC acid phenoxyl radicals that explains the occurrence of *trans*-HC acid and aldehyde links in the chains of natural lignins.

**Table 5:** Geometry parameters of HC acid molecules and corresponding radicals and radical cations in various states of ionization. Atom numbering according to Figure 1.

Phenolic Acid	State of Ionization	D (°)		Length of the Bond (Å)			
		(4,3,7,8)	(2,3,7,8)	O–H	C_6_–O	C_1_–O	C_5_–O
*cis*-*p*-Coumaric	*z* = 0	159.35	24.14	0.96	1.36	–	–
	*z* = −1	−167.98	13.06	0.96	1.39	–	–
	*z* = −2	180.00	0.01	–	1.28	–	–
	radical, *z* = 0	−161.99	20.29	–	1.24	–	–
	radical, *z* = −1	−96.46	94.99	–	1.26	–	–
	radical cation, *z* = − 1	−11.80	169.21	0.97	1.32	–	–
	radical cation, *z* = 0	−8.21	172.39	0.96	1.36	–	–
*trans*-*p*-Coumaric	*z* = 0	179.99	−0.01	0.96	1.36	–	–
	*z* = −1	176.18	−3.89	0.96	1.38	–	–
	*z* = −2	−180.01	−0.01	–	1.28	–	–
	radical, *z* = 0	−180.02	−0.01	–	1.24	–	–
	radical, *z* = −1	178.09	−1.60	–	1.25	–	–
	radical cation, *z* = − 1	−179.99	0.01	0.97	1.32	–	–
	radical cation, *z* = 0	0.00	−180.00	0.96	1.36	–	–
*cis-*Ferulic	*z* = 0	−38.48	144.81	0.96	1.36	1.37	–
	*z* = −1	−12.28	168.97	0.97	1.38	1.39	–
	*z* = −2	0.71	−179.02	–	1.28	1.40	–
	radical, *z* = 0	−153.78	28.62	–	1.24	1.33	–
	radical cation, *z* = 0	−7.05	174.42	–	1.26	1.37	–
	radical cation, *z* = − 1	−13.65	167.82	0.97	1.32	1.32	–
*trans-*Ferulic	*z* = 0	−180.03	−0.02	0.97	1.36	–	1.37
	*z* = −1	179.66	−0.06	0.97	1.37	–	1.38
	*z* = −2	179.69	0.08	–	1.28	–	1.40
	radical, *z* = 0	−0.01	179.99	–	1.23	–	1.34
	radical cation, *z* = 0	180.00	−0.00	0.97	1.35	–	1.37
*cis-*Sinapic	*z* = 0	−33.83	148.72	0.97	1.36	1.37	1.37
	*z* = −1	−11.67	169.50	0.97	1.38	1.39	1.38
	*z* = −2	2.01	−177.59	–	1.28	1.40	1.40
	radical, *z* = 0	−24.99	156.92	–	1.24	1.34	1.34
	radical, *z* = −1	−9.20	172,50	–	1.25	1.37	1.35
	radical cation, *z* = −1	−13.75	167.28	0.98	1.31	1.32	1.34
	radical cation, *z* = 0	−140.16	42.29	0.97	1.36	1.36	1.37
*trans-*Sinapic	*z* = 0	0.01	180.01	0.97	1.35	1.36	1.37
	*z* = −1	−7.10	172.93	0.97	1.37	1.37	1.38
	*z* = −2	178.44	−0.53	–	1.28	1.40	1.40
	radical, *z* = −1	176.02	−2.91	–	1.25	1.36	1.37
	radical cation, *z* = −1	−180.00	−0.01	0.98	1.31	1.32	1.34
	radical cation, *z* = 0	−4.02	175.93	0.97	1.35	1.36	1.37

### 2.6. Bond Lengths and Stabilization of the Phenoxyl Radical

To assess the possibilities of hydrogen bonds formation, which stabilizes phenoxyl cation–radicals, the lengths of O–H, C_6_–O, C_1_–O/C_5_–O bonds were calculated based on the optimized geometry of the molecules (Table 5). Although the changes in bond lengths were insufficient to make firm conclusions, an overall trend of 0.1 Å increase in phenolic O–H bond length was observed for radical cation forms of the studied acids, which might mean the formation of hydrogen bond with oxygen atom of the neighboring oxygen containing groups.

### 2.7. Energy Descriptors and Antioxidant Capacity

For further analysis of the antioxidant action mechanisms, the corresponding sets of parameters were calculated for HB and HC acids in different states of ionization:

(1) The mechanism of direct hydrogen atom transfer (HAT): bond dissociation enthalpy (BDE) for O–H of the phenolic hydroxyl.
(2) The mechanism of single electron transfer followed by proton transfer (SET–PT): ionization potential (IP) and the proton dissociation enthalpy (PDE).
(3) The mechanism of sequential proton loss and electron transfer (SPLET): proton affinity (PA) and electron transfer enthalpy (ETE).

The IP values were calculated on the basis of the data on molecular orbital energies (IPo) and the total energy of the molecules (IPe), according to Equations (6) and (7). The values of ETE, PA, and PDE were calculated for vacuum at a temperature of 298K with subtraction of proton (H^+^) and electron formation enthalpy. The obtained values are not the absolute values of the thermodynamic parameters considered, but they can be properly used to compare different HB and HC acids and reveal the correlation relationships in the context of the present work. A similar approach was used when characterizing the antioxidant properties of flavonoids quercetin and myricetin [20]. The calculated thermodynamic and energy parameters for HB and HC acid molecules are presented in Table 6a,b.

*IP_O_* = −*E_HOMO_* (6)

*IP_E_* = (*ε_o_* + *E_tot_*)_*A*^⊕^_ − (*ε*_0_ + *E_tot_*)_*A*_ (7)

where: E_HOMO_—energy of the highest occupied molecular orbital; (*ε_o_* + *E_tot_*)—total energy for the molecule and cation radical of the antioxidant, respectively.

For non-ionised form of HB and HC acids the IPe values range from 7.27 eV (*cis*-SinA) to 8.62 eV (*p*-HBA). The typical IP values in vacuum for the phenolic compounds in non-ionised form are ≤9 eV [56]. For monoanion forms of HB and HC acids the calculated IPe values range from 3.33 to 3.85 eV (Table 6a). The obtained IPe values for monoanion forms of HB and HC acids are 1.2–1.3 times lower than those found in literature for *p*-HBA (4.49 eV), GalA (4.44 eV), *trans*-CA (4.32 eV), *trans*-FA (4.89 eV) and *trans*-SinA (4.51 eV) [47,53]. These differences emerge due to the features of semiempirical calculation methods in the framework of the density functional theory [57].

The data in Table 6b shows that the PDE of phenolic hydroxyl O-H in non-ionised forms of HB and HC acids decreases in the row: *p*-HBA (86.55 kcal/mol) > VA (85.76 kcal/mol) > *trans*-CA (82.54 kcal/mol) > GalA (82.35 kcal/mol) > *trans*-FA (82.14 kcal/mol) > SyrA (81.65 kcal/mol) > *trans*-SinA (78.57 kcal/mol), which agrees with the data in other works [58,59]. It should also be noted that the calculated O–H bond BDE in the gas phase for non-ionized forms of HB and HC acids fits within the range of values of the same thermodynamic parameter (75.78–81.41 kcal/mol) for widely known natural antioxidants—tocopherols [60].

**Figure 7:**
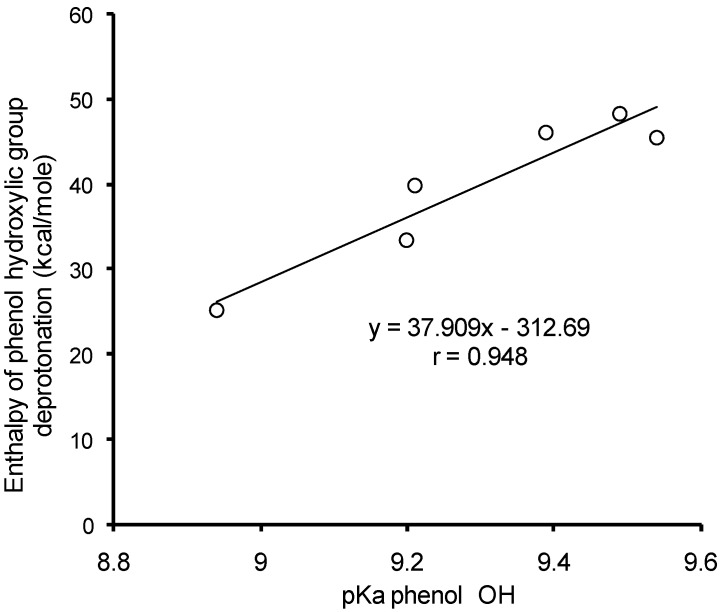
Correlation analysis of deprotonation enthalpy *versus* pKa values of phenyl hydroxyls of mono-HB and mono-HC acids.

Based on the values of enthalpy of formation for monoanion and dianion forms of HB and HC acids, the enthalpies of phenyl hydroxyl deprotonation have been calculated. The enthalpy of H^+^ formation was 1530.17 kJ/mol [20]. The comparison of calculated values for enthalpy of phenyl hydroxyl deprotonation to the experimental pKa values for mono-HB and mono-HC acids in aqueous solutions [61] revealed a significant (*p* < 0.05) correlation (*r* = 0.948) (Figure 7).

**Table 6:** (**a**,**b**) Thermodynamic and energy parameters of HB and HC acids in various states of ionization in the gas phase (298 K).

**(a)**						
**Phenolic Acid**	**BDE, kcal/mol**	**IPe, eV**	**IPo, eV**	**E_HOMO_, eV**	**E_LUMO_, eV**	**χ, eV**
*p-*OH Benzoic						
*z* = 0	86.55	8.62	6.86	−6.866	−1.576	4.221
*z* = −1	72.95	3.42	1.63	−1.627	1.884	−0.129
*z* = −2	–	−1.11	−2.76	3.757	5.421	−4.089
Vanillic						
*z* = 0	85.76	8.04	6.41	−6.413	−1.527	3.970
*z* = −1	74.83	3.43	1.68	−1.679	1.873	−0.097
*z* = −2	–	−1.05	−2.55	2.554	4.587	−3.570
Siringic						
*z* = 0	81.65	7.66	6.12	−6.121	−1.485	3.803
*z* = −1	73.21	3.40	1.69	−1.691	1.906	−0.107
*z* = −2	–	−1.01	−2.36	2.363	4.478	−3.421
Gallic						
*z* = 0, C_1_	82.35	8.06	6.41	−6.405	−1.645	4.025
*z* = 0, C_6_	82.86	8.06	6.41	−6.405	−1.645	4.025
*z* = 0, C_5_	77.97	8.06	6.41	−6.405	−1.645	4.025
*z* = −1, C_1_	75.70	3.57	1.83	−1.827	1.933	−0.053
*z* = −1, C_6_	73.26	3.57	1.83	−1.827	1.933	−0.053
*z* = −1, C_5_	69.97	3.57	1.83	−1.827	1.933	−0.053
*z* = −2, C_1_	–	−0.68	−2.56	2.580	4.923	−3.571
*z* = −2, C_6_	–	−0.74	−2.65	2.651	5.064	−3.858
*z* = −2, C_5_	–	−0.93	−2.58	2.580	4.293	3.751
*cis*-Caffeic						
*z* = 0	87.06	8.12	6.41	−6.413	−2.119	4.266
*z* = −1	77.73	3.44	1.74	−1.736	1.831	−0.047
*z* = −2	–	−0.44	−2.18	2.175	4.775	−3.475
*trans*-Caffeic						
*z* = 0	82.54	8.00	6.43	−6.435	−2.150	4.292
*z* = −1	67.80	3.33	1.58	−1.581	1.429	0.076
*z* = −2	–	−0.52	−1.97	1.968	4.873	−3.420
*cis*-Ferulic						
*z* = 0	77.46	7.53	6.65	−6.646	−2.105	4.376
*z* = −1	76.03	3.44	1.79	−1.787	1.673	0.051
*z* = −2	–	−0.54	−2.02	2.022	4.210	−3.117
*trans*-Ferulic						
*z* = 0	82.14	7.66	6.14	−6.144	−2.100	4.122
*z* = −1	82.64	3.85	1.54	−1.537	1.615	−0.038
*z* = −2	–	−0.44	−1.82	1.822	4.172	−2.997
*cis*-Sinapic						
*z* = 0	78.92	7.27	6.35	−6.347	−2.089	4.218
*z* = −1	70.20	3.61	1.89	−1.890	−1.816	0.037
*z* = −2	–	−0.80	−1.88	1.885	4.144	−3.014
*trans*-Sinapic						
*z* = 0	78.57	7.38	5.94	−5.940	−2.082	4.011
*z* = −1	69.25	3.58	1.64	−1.635	1.541	0.047
*z* = −2	–	−0.38	−1.68	–	4.085	−2.882
**(b)**						
**Phenolic Acid**	**η, eV**	**ω × 10^3^, eV**	**E_HOMO_–E_LUMO_, eV**	**ETE, kJ/mol**	**PA, kJ/mol**	**PDE, kJ/mol**
*p*-OH Benzoic						
*z* = 0	2.645	842	5.290	–	–	838.5
*z* = −1	1.755	1.179	3.511	−106.87	1720.5	1283.3
*z* = −2	1.131	1569	2.664	–	–	–
Vanillic						
*z* = 0	2.443	806	4.887	–	–	891.5
*z* = −1	1.776	0.658	3.552	−101.69	1723.1	1290.9
*z* = −2	1.017	1567	2.033	–	–	–
Siringic						
*z* = 0	2.318	780	4.636	–	–	910.0
*z* = −1	1.798	0.800	3.597	−97.85	1260.4	834.9
*z* = −2	1.058	1383	2.116	–	–	–
Gallic						
*z* = 0, C_1_	2.380	851	4.760	–	–	875.3
*z* = 0, C_6_	2.380	851	4.760	–	–	877.4
*z* = 0, C_5_	2.380	851	4.760	–	–	856.9
*z* = −1, C_1_	1.880	0.188	3.760	−65.99	1691.1	1281.0
*z* = −1, C_6_	1.880	0.188	3.760	−71.05	1686.0	1270.8
*z* = −1, C_5_	1.880	0.188	3.760	−89.97	1691.1	1257.0
*z* = −2, C_1_	1.172	1501	2.343	–	–	–
*z* = −2, C_6_	1.207	1541	2.414	–	–	–
*z* = −2, C_5_	1.171	1501	2.343	–	–	–
*cis*-Caffeic						
*z* = 0	2.147	1060	4.294	–	–	–
*z* = −1	1.783	0.156	3.567	−42.30	1675.9	889.4
*z* = −2	1.300	1161	2.600	–	–	1301.3
*trans*-Caffeic						
*z* = 0	2.143	10.75	4.285	–	–	–
*z* = −1	1.505	0.481	3.010	−49.82	1641.8	881.7
*z* = −2	1.452	1007	2.905	–	–	1270.6
*cis*-Ferulic						
*z* = 0	2.270	1054	4.541	–	–	–
*z* = −1	1.730	0.232	3.460	−42.27	1656.2	906.1
*z* = −2	1.094	1109	2.188	–	–	1141.3
*trans*-Ferulic						
*z* = 0	2.022	1050	4.044	–	–	–
*z* = −1	1.576	0.119	3.152	−42.45	1696.6	913.4
*z* = −2	1.175	955	2.350	–	–	1283.0
*cis*-Sinapic						
*z* = 0	2.129	1044	4.258	–	–	–
*z* = −1	1.853	0.091	3.706	−77.64	1679.7	936.9
*z* = −2	1.130	1006	2.259	–	–	1253.5
*trans*-Sinapic						
*z* = 0	1.929	1043	3.858	–	–	925.2
*z* = −1	1.588	0.071	3.176	−37.04	1635.2	1253.1
*z* = −2	1.203	863	2.405	–	–	–

**Figure 8:**
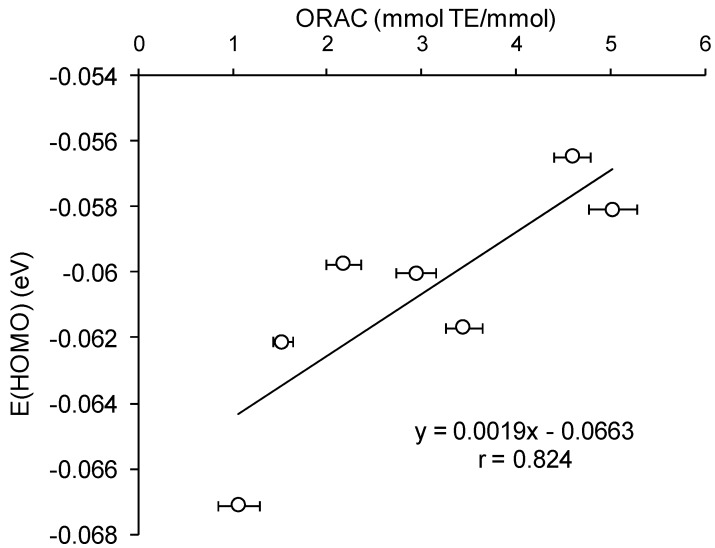
Correlation analysis of AOC against peroxyl radical for HB and HC acids *versus* HOMO energy values.

**Figure 9:**
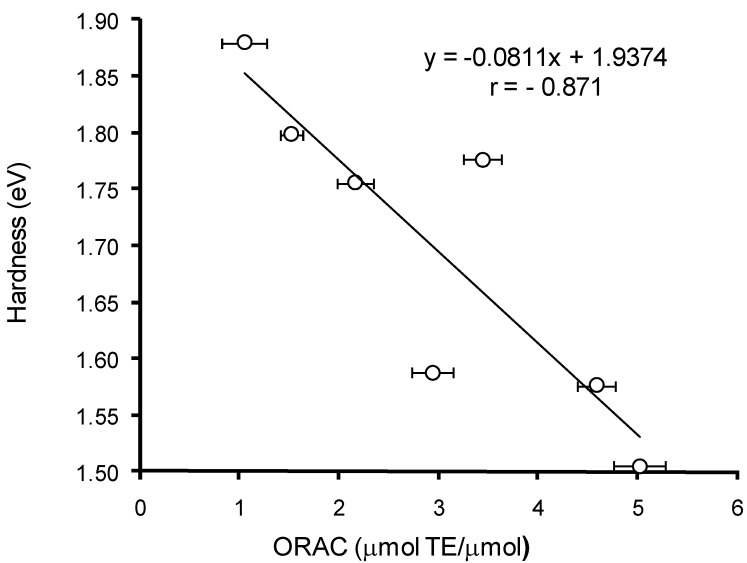
Correlation analysis of AOC against peroxyl radical for HB and HC acids *versus* rigidity (η).

The comparative analysis of the AOC of HB and HC acids against peroxyl radical (Table 2) *versus* calculated quantum-chemical descriptors of the hydroxy-aromatic acids (Table 6a,b) has revealed a significant (*p* < 0.05) correlation between the values of AOC, energy of HOMO (*r* = 0.824), and rigidity (*r* = −0.871) for monoanion forms of these compounds (Figure 8 and Figure 9). Thus, the lower values of AOC against peroxyl radical for HB acids compared to HC acids can be explained by their higher HOMO energy and rigidity.

Since AOC of HB and HC acids is largely dependent on pH of reaction medium (Figure 2), the understanding of mechanisms for their antioxidant action required to analyse how thermodynamic and energy parameters of HB and HC acids depend on their state of ionization. The analysis of the data in Table 6a,b indicates that carboxyl group deprotonation in GB and GC acids leads to the 1.1 times reduction of O–H bond BDE, the 2.2 times IPe reduction, and also the 1.3 times reduction of rigidity and difference between LUMO and HOMO energies. In addition, the carboxyl group deprotonation in HB and HC acids increases the HOMO energy and reduces the electronegativity of the molecules. Altogether, the above changes in energy and thermodynamic parameters indicate a significantly lower stability of the monoanion form of HB and HC acids compared to the non-ionised form, therefore, the carboxyl group deprotonation should result in greater reactivity of HB and HC acids. The last assumption is fully confirmed experimentally by the significant AOC increase of HB and HC acids in the pH range corresponding to the ionization change of carboxyl group (Figure 2).

Further phenolic hydroxyl deprotonation and phenolate-anion formation leads to a more severe destabilization of HB and HC acid molecules—the HOMO energy reaches positive values whereas the IPe values drop significantly (Table 6a). The rigidity of the molecules and the difference between LUMO and HOMO energies reduce 2.2 times compared to the non-ionized form of HB and HC acids. The negative IPe values of the phenolate anions of HB and HC acids indicate their high electron-donating abilities. Therefore, even the smallest quantities of HB and HC acids present in the solution in phenolate form will very quickly eliminate due to the interaction with oxidizing agents, with a result of continuous shift of chemical equilibrium in the reaction of dissociation of phenolic hydroxyl in HB and HC acids. Thus, the most likely mechanism of interaction between HB/HC acids and ABTS radical cation at pH > 4.5 appears to be sequential proton loss and electron transfer (SPLET).

**Figure 10:**
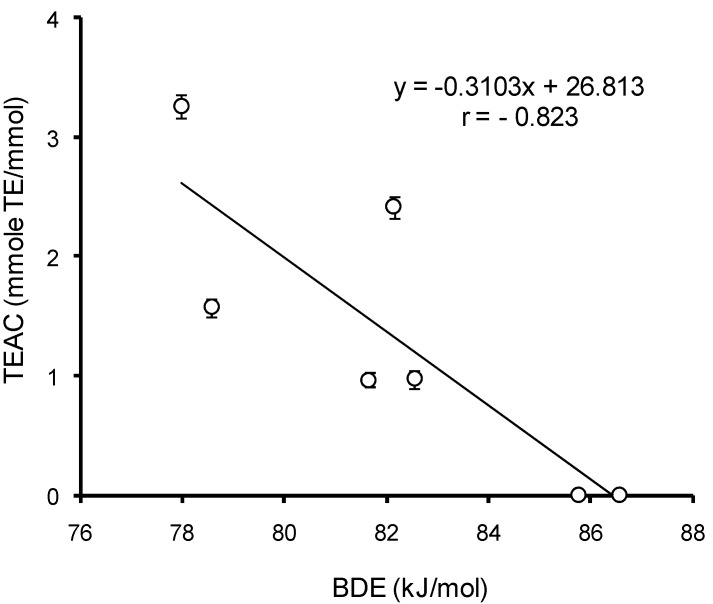
The correlation analysis of HB/HC acid AOC against the ABTS cation–radical at pH 3.5 *versus* values of bond dissociation enthalpy (BDE) in phenolic hydroxyl.

**Figure 11:**
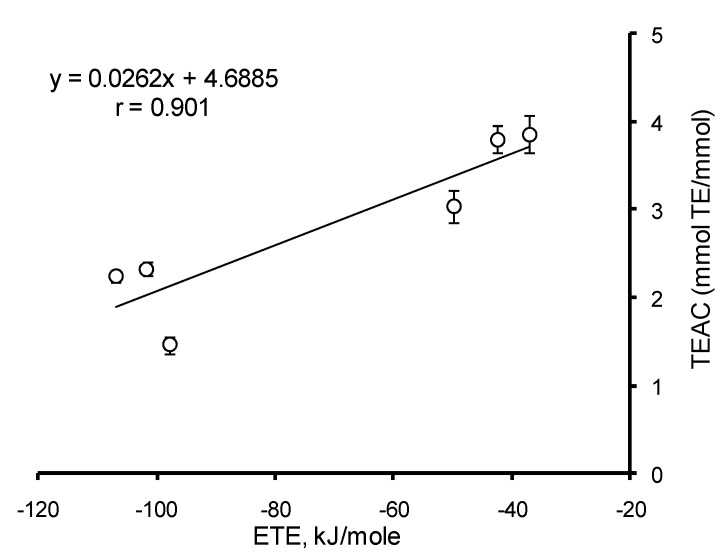
Correlation analysis of HB/HC acid AOC against the ABTS cation radical at pH 7.4 *versus* values of electron transfer enthalpy (ETE) from phenolate ion.

To identify the mechanism of interaction between HB/HC acids and ABTS radical cation, the correlation analysis of AOC with the thermodynamic parameters of uncharged and monoanion forms of HB and HC acids was carried out at different pH. For the uncharged forms, among all parameters a significant (*p* < 0.05) correlation (*r* = −0.832) was established between BDE values and AOC of HB and HC acids against the ABTS cation–radical at pH 3.5 (Figure 10). This means that the dominating mechanism of interaction of HB and HC acids with ABTS radical cation at pH ≤ 3.5 is hydrogen atom transfer (HAT). In the range of pH 4.0–7.5 the only value to significantly (*p* < 0.05) correlate with AOC of mono-HB and mono-HC acids against the ABTS radical cation is ETE (*r* = 0.901) (Figure 11). Consequently, when the pH of the reaction medium is 4.0–7.5, the main mechanism of implementation of the antioxidant properties of HB and HC acids against the ABTS cation–radical becomes sequential proton loss and electron transfer (SPLET). Thus, the mechanism of antioxidant action of HB and HC acids appears to depend on pH.

To resolve the question of antioxidant mechanism, previously the quantitative criteria were proposed based on comparison of IP and BDE of various phenolic substances with phenol [19]: for absolute values of ΔIP up to 36 kcal/mol and for ΔBDE ~−10 kcal/mol the mechanism is dominated by hydrogen atom transfer, whereas for ΔIP > 45 kcal/mol the mechanism is predominantly SET. Based on the data in Table 6a,b and values of BDE and IP of phenol from [62,63], the values of ΔIP and ΔBDE were calculated for HB and HC acids. For uncharged forms of HB and HC acids the values of ΔIP and ΔBDE lay in ranges of (−12.82)–(−4.03) and (−28.58)–(+2.53) kcal/mol, respectively, which confirms the earlier assumption about the prevalence of HAT mechanism of the antioxidant properties implementation of HB and HC acids at strongly acidic pH values, where ionization of carboxyl group does not occur. For monoanion forms of all investigated HB and HC acids the absolute values of ΔIP exceeded 100 kcal/mol, which favors the implementation of the antioxidant properties via SPLET mechanism. As it was indicated above, the electron transfer becomes possible after the deprotonation of phenolic hydroxyl.

Given that *p*-HB and *p*-HC acids are structural analogs of tyrosine, the interaction of tyrosine-containing dipeptides with the ABTS radical cation at pH 7.4 should be expected to follow the same mechanism of sequential proton loss and electron transfer (SPLET).

## 3. Experimental Section

### 3.1. Chemicals

Vanillic acid, *p*-hydroxybenzoic acid, syringic acid, gallic acid, *p*-coumaric acid, ferulic acid, sinapic acid were purchased from Sigma-Aldrich (St. Louis, MO, USA). Fluorescein sodium salt, potassium peroxodisulfate, 2,2'-azino-bis-(3-ethylbenzothiazoline-6-sulphonic acid) diammonium salt (ABTS), and 6-hydroxy-2,5,7,8-tetramethylchroman-2-carboxylic acid (Trolox), standards of d- and l-amino acids, *N*-acetyl-l-tryptophanamide, methyl-β-cyclodextrin were purchased from Sigma-Aldrich (St. Louis, MO, USA). 2,2'-azobis-(amidinopropane) dihydrochloride (AAPH) was obtained from Acros Organics (Geel, Belgium). All solutions were prepared in 18 MΩ·cm^−1^ water. Other reagents were of at least analytical grade.

### 3.2. Analysis of Antioxidant Capacity of Phenolic Acids

For determination of AOC the hydroxyaromatic acids were diluted in deionised water. Since the AOC value depends on the concentration of the test antioxidant in the reaction medium, for each test compound the concentration range was determined in which the dependence of the antioxidant effect on the antioxidant concentration is described by a linear function with high reliability (the coefficient of determination *R*^2^ > 0.99).

For testing of the AOC against the ABTS radical cation, the optimum concentration range was 3–12 µM for GalA; 10–50 µM for SyrA, VA and *p*-HBA; and 5–20 µM for hydroxycinnamic acids. For testing of the AOC of the phenol carbonic acids against peroxyl radical the optimum concentration range was 3–15 µM for *p*-HBA, VA, CA and FA; 5–25 µM for SinA and SyrA; and 20–60 µM for GalA.

#### 3.2.1. Analysis of Antioxidant Capacity against ABTS Radical Cation

The radicals were generated according to Re *et al.* [32]. ABTS and potassium peroxodisulfate were dissolved in water and incubated in the dark at final concentrations of 7 and 2.45 mM, respectively, at ambient temperature for 12–16 h. The ABTS·^+^ stock solution was further diluted with phosphate buffer saline (PBS, 100 mM sodium chloride, 50 mM potassium dihydrogenphosphate, pH 7.40) to get the optical density 0.70 ± 0.02 at 734 nm, which corresponds to ABTS·^+^ concentration ~48 µM (ε = 1.5 ° 10^4^ L/mol ° cm). Trolox solutions (10–100 µM) were used for calibration. The reaction was initiated by mixing 20 µL of studied acid solution with 180 µL of ABTS·^+^, followed by measurement of the absorbance at 734 nm for 40 min using Synergy 2 plate reader (BioTek, Winooski, VT, USA). All measurements were performed in 4 replicates. TEAC values were calculated based on the linear regression equation between the trolox concentration and the decrease in the absorbance of ABTS·^+^ (Δ*D* = *D*_blank_ − *D*_standard_) (Δ*D* = 0.0012[Trolox]). AOC was expressed as amount of Trolox μmolar equivalents (TE, µM) per µM of antioxidant.

#### 3.2.2. Determination pH-Dependence of AOC for Phenolic Acids against ABTS Radical Cation

AOC of hydroxyaromatic acids was studied in pH range of 3.5 to 8.0 in universal buffer (0.04 M ortophosphoric acid, 0.04 M acetic acids, 0.04 M boric acid). pH values of universal buffer were adjusted with 0.2 M NaOH.

#### 3.2.3. Determination of Antioxidant Capacity by ORAC-FL Assay

ORAC-FL assay is based on the method of Ou *et al*. [64] with modification of Moore *et al*. [65] and Pior *et al*. [66]. The reaction mixture contained 115 µL of fluorescein sodium salt solution (8.16 ° 10^−8^ M) and 15 µL of phenolic acids (83–125 µg/mL) or Trolox (5–75 µM as a standard). The reaction was initiated with addition of 15 µL of 0.6 M AAPH solution [66] and reaction mixture was incubated for 30 s with orbital shaking at 1400 rpm and 37 °C. After incubation the fluorescence intensity was monitored every 60 s for 1 h at two wavelengths—485 nm for excitation and 528 nm for emission—on Synergy 2 plate reader (BioTek, Winooski, VT, USA) at 37 °C. The final ORAC-FL values were calculated using the linear regression equation between the trolox concentration and the net area (net AUC) of the fluorescein decay curve (net AUC = *k*[Trolox] + b) and expressed in µmol TE per µmol of antioxidant. All solutions were prepared in 75 mM sodium phosphate buffer, pH 7.40. The assay was performed in 4 replicates for each sample or standard solution.

### 3.3. Computation of Molecular Geometry and Electron Descriptors of Studied Antioxidants by Semi-Empirical Quantum-Mechanical Methods

Quantum-chemical computations of molecular geometry and electron descriptors of aromatic hydroxyacids were performed using Gaussian 3.0 software package (Gaussian., Inc., Wallingford, CT, USA) at the DFT level of theory (B3LYP hybrid functional) together with 6-31++G**(d,p) basis set. For computation of electron descriptors and geometry of molecules and ions were carried out using restricted basis, for radicals–unrestricted basis. Optimization of molecular geometry was carried out with use of Berny algorithm. After molecular geometry optimization the type of the found stationary point was identified. The absence of negative frequency in oscillation spectrum of atoms indicated a local minimum. Computations were performed: for hydroxyaromatic acids in different states of ionization: *z* = 0 (protonated form), *z* = −1 (deprotonated carboxyl group), *z* = −2 (deprotonated carboxyl group and phenolic hydroxyl); for phenoxyl radical cation (POH·^+^) in ionization state *z* = 0 and *z* = −1; for phenoxyl radicals (PO·) in ionization state *z* = 0, *z* = −1, *z* = −2.

For optimized structures the following electronic and molecular descriptors were calculated: Mulliken spin density distribution; the energy of the highest occupide molecular orbital (HOMO, E_HOMO_) and lower unoccupied molecular orbital (LUMO, E_LUMO_); the difference between HOMO and LUMO energies (E_HOMO_–E_LUMO_); bond dissociation energy in the proton donor group (BDE); ionization potential (IP); electronegativity (γ); electrophilicity (ω); rigidity (η); proton affinity (PA); proton dissociation enthalpy (PDE); electron transfer enthalpy (ETE).

Bond dissociation energy in the proton donor group was calculated as difference between sum of enthalpies of formation for reaction products (antioxidant radical A, proton H·) and enthalpy of formation for the antioxidant (A), according to Equation (8).

*BDE*=((*ε*_0_ + *H_corr_*)_*A*^·^_ + (*ε*_0_ + *H_corr_*)_*H*^·^_−(*ε*_0_ + *H_corr_*)_*A*_)×627.5095 (8)

where: BDE—bond dissociation energy in proton donor group; (*ε*_0_ + *H_corr_*)—enthalpy of formation of corresponding compounds; 627.5095—conversion coefficient of Hartry nuclear units to kcal/mol. Heat of proton formation was −217.99 kJ/mol [20,67].

Ionization potentials IP_O_ were calculated from the molecular orbital energy according to Equation (6), ionization potential IP_E_—from the energetic parameters of the molecule and cation radical of the antioxidant [20], according to Equation (7).

Electron affinity was calculated as EA = −E_LUMO_.

Electronegativity (γ) and rigidity (η) were calculated as differences between HOMO and LUMO (Equations (9) and (10)) [68].

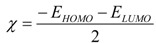
(9)

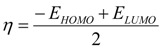
(10)


Electrophilicity (ω) of antioxidant molecules was calculated according to Equation (11).

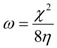
(11)


Proton affinity was calculated as difference between enthalpy of formation for phenolate antioxidant’s anion (*A^−^*) and enthalpy of formation for the antioxidant (*A*) according to Equation (12).

*PA*=((*ε*_0_ + *H_corr_*)_*A*^−^_ − (*ε*_0_ + *H_corr_*)_*A*_)×627.5095×4.1868 (12)

where: PA—proton affinity, kJ/mol; (*ε*_0_ + *H_corr_*)—enthalpy of formation of the corresponding compounds; 627.5095—conversion coefficient of Hartry units to kkal/mol, 4.1868—conversion coefficient from kcal to kJ.

The electron transfer enthalpy was calculated as difference between enthalpies of formation for the phenoxyl radical (*A^.^*) and fenolate anion (*A^−^*), according to Equation (13).

*PEP*=((*ε*_0_ + *H_corr_*)_*A*^.^_ − (*ε*_0_ + *H_corr_*)_*A*^−^_)×627.5095×4.1868 (13)

The proton dissociation enthalpy was calculated as difference of enthalpies of formation for phenoxyl radical (*A*^.^) and phenoxyl cation–radical (*A*^+.^) according to Equation (14).

*PDP*=((*ε*_0_ + *H_corr_*)_*A*^.^_ − (*ε*_0_ + *H_corr_*)_*A*^+.^_)×627.5095×4.1868 (14)

## 4. Conclusions

(1) The structural–functional analysis carried out for *p*-hydroxybenzoic and *p*-hydroxycinnamic acids has revealed that the most significant descriptors of their antioxidant properties against peroxyl radical are HOMO energy, rigidity (η) and Mulliken charge on the carbon atom in *m*-position to the phenolic hydroxyl.

(2) The most significant descriptor of the antioxidant properties against the ABTS radical cation at pH 7.40 is electron transfer enthalpy from the phenolate ion.

(3) Based on the results of quantum-chemical computation, the electron density distribution, geometry and energy parameters of the studied compounds were calculated. The correlations between the calculated parameters and antioxidant capacity at different pH allowed to propose the mechanism of phenyl carbonic acids interaction with the peroxyl radical (hydrogen atom transfer) and the ABTS cation radical (sequential proton loss and electron transfer at pH > 4.5 and hydrogen atom transfer in acidic medium).
